# Flexible and Integrated Sensing Platform of Acoustic Waves and Metamaterials based on Polyimide-Coated Woven Carbon Fibers

**DOI:** 10.1021/acssensors.0c00948

**Published:** 2020-07-20

**Authors:** Ran Tao, Shahrzad Zahertar, Hamdi Torun, Yi Ru Liu, Meng Wang, Yuchao Lu, Jing Ting Luo, Jethro Vernon, Richard Binns, Yang He, Kai Tao, Qiang Wu, Hong Long Chang, Yong Qing Fu

**Affiliations:** † Shenzhen Key Laboratory of Advanced Thin Films and Applications, College of Physics and Optoelectronic Engineering, Shenzhen University, Shenzhen 518060, P. R. China; ‡ Faculty of Engineering and Environment, Northumbria University, Newcastle upon Tyne NE1 8ST, U.K.; § China-EU Institute for Clean and Renewable Energy, Huazhong University of Science and Technology, Wuhan 430074, P. R. China; ∥ Key Laboratory of Micro and Nano Systems for Aerospace, Ministry of Education, Northwestern Polytechnical University, Xi’an 710072, P. R. China

**Keywords:** surface acoustic wave, carbon fiber, electromagnetic metamaterials, biosensors, microfabrication

## Abstract

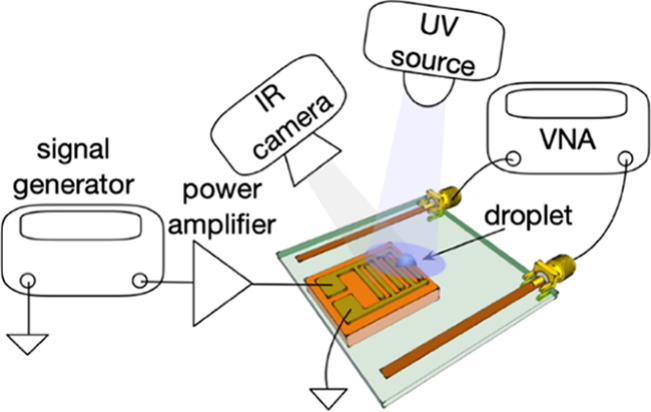

Versatile, in situ sensing and continuous monitoring capabilities are critically needed, but challenging, for components made of solid woven carbon fibers in aerospace, electronics, and medical applications. In this work, we proposed a unique concept of integrated sensing technology on woven carbon fibers through integration of thin-film surface acoustic wave (SAW) technology and electromagnetic metamaterials, with capabilities of noninvasive, in situ, and continuous monitoring of environmental parameters and biomolecules wirelessly. First, we fabricated composite materials using a three-layer composite design, in which the woven carbon fiber cloth was first coated with a polyimide (PI) layer followed by a layer of ZnO film. Integrated SAW and metamaterials devices were then fabricated on this composite structure. The temperature of the functional area of the device could be controlled precisely using the SAW devices, which could provide a proper incubation environment for biosampling processes. As an ultraviolet light sensor, the SAW device could achieve a good sensitivity of 56.86 ppm/(mW/cm^2^). On the same integrated platform, an electromagnetic resonator based on the metamaterials was demonstrated to work as a glucose concentration monitor with a sensitivity of 0.34 MHz/(mg/dL).

Solid woven carbon fibers are widely used in various fields such as aerospace,^1^ electronics,^2,3^ and medical transducers,^4^ where low weight, high stiffness, and high conductivity are critically required. For these applications, in situ, versatile sensing and continuous monitoring capabilities are often required. For example, built-in sensors are often required for monitoring structural health of composite aircrafts made of woven carbon fibers^5^ to detect crack generation and propagation in these structures.^6^ However, currently few studies are focused on new types of applications using carbon fiber-based composites for various environmental applications such as temperature and ultraviolet (UV) light sensing or biological applications such as biomolecular and biochemical sensing. For these applications, a key challenge is to develop an integrated approach with the capabilities of efficient biosampling, liquid actuation, high-precision detection, and wireless operation/monitoring capabilities.

Surface acoustic wave (SAW) devices including those thin-film ones based on ZnO and AlN have been extensively explored for a wide range of applications including gas sensing,^7,8^ environmental sensing,^9,10^ biomolecular detection,^11,12^ microfluidics,^13−15^ acoustic tweezers,^16,17^ and lab-on-a-chip.^18,19^ SAW sensors have the capability to be developed into a wireless operation platform, which can be realized by integrating antennas to the electrodes for signal transmission.^20,21^ Alternatively, a new approach of utilizing the same SAW structure as an electromagnetic resonator or metamaterials has been introduced recently.^22^ This is based on defining an electromagnetic metamaterial-based resonator on the SAW device structure, which can be excited using external antennas.^23^ It allows a new mode of sensing based on subwavelength-sized structures defined by the SAW geometries that are usually made of metals on dielectric substrates, and the changes of electromagnetic resonant frequencies of this structure can be applied to monitor parameters of interest for sensing applications.^22^ Using this new design, the operation using metamaterials can be utilized in addition to the conventional operation of SAWs for sensing or acoustofluidics, where the interdigitated transducers (IDTs) are powered directly and remotely.

In this study, we explored a new concept of integrated sensing technology on woven carbon fibers through the integration of electromagnetic metamaterials and thin-film acoustic wave sensors, with capabilities of noninvasive, in situ, and continuous monitoring of environmental parameters and biomolecules wirelessly. It is well known that the woven structure of carbon fibers poses challenges to define efficient SAW and electromagnetic resonators due to its highly flexible, extremely porous, and rough surface, which causes significant difficulties in coating uniform piezoelectric layers such as ZnO. In addition to mechanical imperfections, the porosity and flexibility of the woven structure could lead to significant damping and reduction of quality factor for both the SAW and metamaterials devices.^24^ We addressed this challenge by fabricating composite materials using a three-layer composite design. The carbon fiber was first coated with a polyimide (PI) layer, and then a ZnO film was deposited onto this PI/carbon fiber structure. We then fabricated SAW and metamaterials devices on this composite material using a conventional photolithography method and optimized the electrodes of the designs for integrated functions including liquid temperature control, UV sensing, and glucose monitoring as case studies for different applications.

## Experimental Section

### Experimental Methods

A ZnO thin film (5 μm thick) was deposited on the PI-coated carbon fiber substrate using a DC magnetron sputter with the sputtering power of 400 W, Ar/O_2_ gas flow rate of 10/15 sccm, and chamber pressure of 4 × 10^–4^ mbar. A zinc target with 99.99% purity was used, while the sample holder was rotated during the deposition to achieve the uniformity of the film thickness. The IDTs were patterned using the conventional photolithography and lift-off process, where Cr/Au films with thicknesses of 10 nm/120 nm were selected as the electrode materials and deposited using a thermal evaporator (EDWARDS AUTO306).

The crystal orientation and surface roughness of the sputtered ZnO thin film were characterized using X-ray diffraction (XRD, SIEMENS D5000) and atomic force microscopy (AFM, Veeco Dimension 3100), respectively. The reflection and transmission spectra of the integrated platform were acquired continuously during the UV- and glucose-sensing experiments using a high-frequency network analyzer (Agilent N5230A) with a LabVIEW data acquisition program. The SAW devices were acoustically excited using a signal generator and a power amplifier while the temperature of the droplet placed on top of the device was recorded using an infrared camera.

### Numerical Methods

The finite element analysis (FEA) simulation of SAWs in this work was performed using the COMSOL software with solid mechanics and electrostatics modules. A two-dimensional (2D) model with a simplified SAW structure was used comprising the carbon fiber layer, PI layer, ZnO thin film, and IDT fingers from bottom to top, with thicknesses of 600 μm, 150 μm, 5 μm, and 130 nm, respectively. The width of the model was defined by the wavelengths of the SAW devices, varying from 64 to 160 μm. The wave modes and reflection spectra S_11_ of SAWs were obtained from the simulation results, with periodic boundary conditions.

The electromagnetic behavior of the coupled device with a wavelength of 64 μm was studied using a commercially available simulator (CST Studio Suite, Darmstadt, Germany). The computational environment was created based on the geometry, and the waveguide ports were defined to obtain scattering parameters. The mesh sizes were refined considering the convergence of the simulations. Plane wave excitations were used during the simulations.

## Results and Discussion

### Design and Characterization of the Integrated Platform

The design of SAW devices relies on the definition of the IDTs so that the device supports specified acoustic wave modes. Rayleigh waves are generated when the IDTs are excited electrically at their resonant frequencies, which are determined by the velocity of sound on the composite structure and the wavelength of the IDT: e.g., *f*_0_ = *v*/λ, where *v* is the acoustic phase velocity and λ is the designed wavelength. Since the phase velocity of piezoelectric materials is altered by different factors, the resonant frequency of the SAW devices can be monitored to track these changes, based on the following relationship^25^
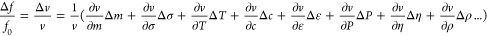
1where *m* is the mass load, σ is the conductivity, *T* is the temperature, *c* is the mechanical constant, ε is the dielectric constant, *P* is the pressure, η is the viscosity, and ρ is the density.

**Figure 1:**
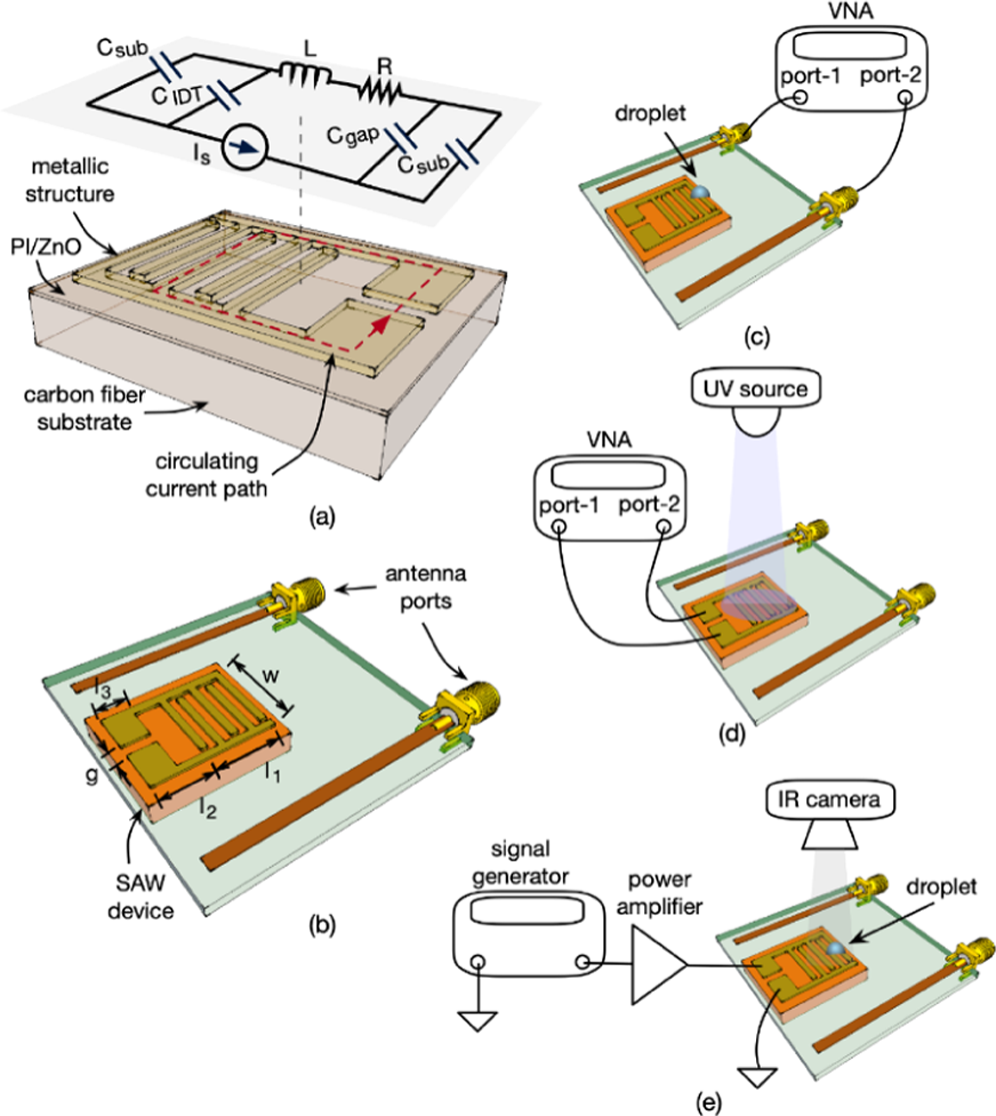
(a) Schematic illustration of the integrated platform combining surface acoustic waves and metamaterials with the equivalent circuit of the device at resonance. (b) Schematic illustration of the integrated platform. Schematic illustrations of the experimental setups for (c) glucose sensing, (d) UV sensing, and (e) temperature control.

Meanwhile, this structure of a single-metallic layer on a dielectric substrate is also an ideal platform to realize a metamaterial-based electromagnetic resonator at microwave frequencies. The structure supports circulating currents along the metallic layer when the device is excited appropriately. For example, when the magnetic field is perpendicular to the device, a circulating current path is generated due to the induced current on the metallic layer as shown in Figure 1a. The induced current can be supported at a specific resonant frequency determined by the geometry of the structure; therefore, its resonant frequency depends on the electrical characteristics imposed by the device geometry. Along the path, the equivalent circuit components can be simplified using lumped elements as labeled in Figure 1a. The resonant frequency and the quality factor of the device can be expressed using eqs 2 and 3.(26)
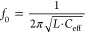
2
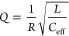
3where *L* is the inductance of the structure, *R* is the equivalent resistance of the structure, and *C*_eff_ is the effective capacitance of the structure. The effective capacitance is determined by the combination of the capacitive elements along the current path including those of the IDTs, gap, and substrate surface. Therefore, any changes in the effective inductance and the capacitance of the structure will alter the resonant frequency of the device. We designed this type of metamaterial device, which is sensitive to the changes in relative permittivity of its substrate and of a sample placed within its vicinity. The changes in the relative permittivity of the device or the sample result in a change in the effective capacitance, thus altering the resonant frequency of the device. The resonant frequency of the device can be simply measured using a pair of monopole patch antennas as shown in Figure 1b.

In this configuration, the sensing structure is electrically passive and electromagnetically coupled to the readout antennas. This eliminates the need for active electronics and power transfer on the sensing structure; therefore, the sensor can be realized in a smaller footprint and consumes negligible power on itself. In comparison, conventional wireless sensing architectures are based on electrically active sensors that are powered using inductively coupled coils.^27,28^

To integrate SAW and metamaterials devices on the woven carbon fiber surfaces, we created a trilayer structure, as shown in Figure 1a. The commercially available woven carbon fiber layer with a thickness of ∼1 mm was coated with a layer of 150 μm thick polyimide (PI) to create a relatively smooth surface for the subsequent processes. Then, a ZnO film layer with a thickness of ∼5 μm was deposited using a DC magnetron sputter. The metallic layer was then patterned on top of the ZnO layer to form the IDTs using a standard lift-off process. The IDTs were made of 20/120 nm thick Cr/Au layers evaporated on the surface. We fabricated devices with different IDT wavelengths of 64, 100, and 160 μm, where the width, length, and gap of the pattern (see Figure 1b) are *w* = 9 mm, *l*_1_ = 5.6 mm, *l*_2_ = 6.2 mm, *l*_3_ = 4 mm, and *g* = 3.2 mm.

**Figure 2:**
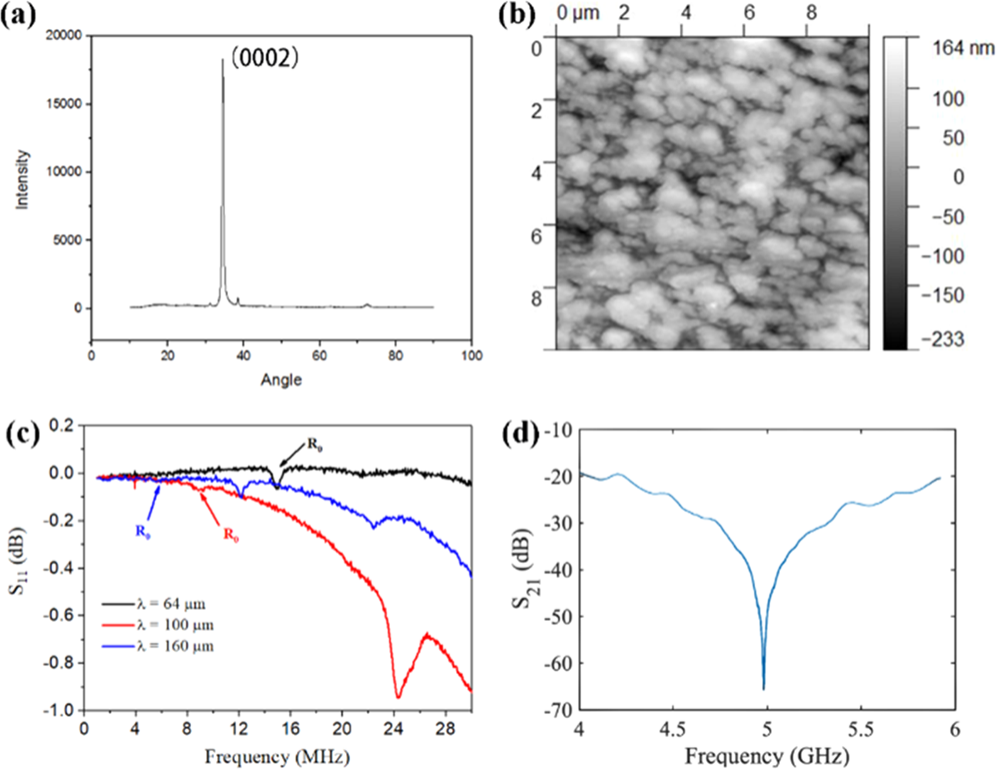
(a) XRD patterns of the ZnO/PI/carbon fiber tri-layer structure. (b) AFM image of the ZnO thin film. (c) Reflection spectra S_11_ of SAWs with the designed wavelengths of 64, 100, and 160 μm. (d) Transmission spectrum S_21_ of the electromagnetic resonator of the SAW device with a wavelength of 64 μm.

Figure 2a shows the XRD pattern of the fabricated tri-layer composite material. There is a dominant peak at 2θ =34°, suggesting that the ZnO film is composed of polycrystalline phases with a strong texture along the *c*-axis (e.g., with strong (0002) orientation). The topographic image of the ZnO film over an area of 10 × 10 μm^2^ obtained using the AFM reveals that its surface roughness is ∼38.6 nm (see Figure 2b).

The reflection spectra S_11_ of SAW devices were measured using a vector network analyzer connected to their electrodes, and the results are shown in Figure 2c. The obtained frequencies of the Rayleigh wave (R_0_) modes are decreased from 14.95 to 5.92 MHz with the wavelength increased from 64 to 160 μm. On the other hand, the electromagnetic resonance of the devices with a wavelength of 64 μm was also characterized, and the results of transmission spectra S_21_ are shown in Figure 2d. The electromagnetic resonant frequency was measured as 4.98 GHz. In this design, the wavelength of the IDT does not alter the resonant frequency as the *C*_eff_ parameter of eq 3 is dominated by the surface capacitance of the structure.

### Acoustic Wave Modes and Electromagnetic Fields

**Figure 3:**
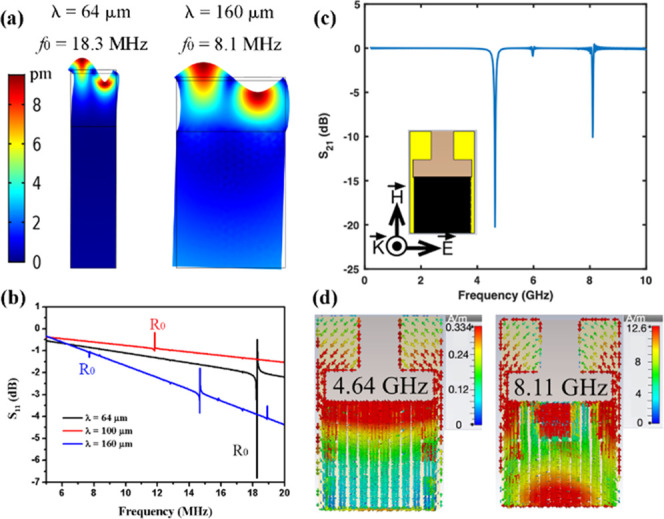
FEA simulation of vibration modes of SAW devices based on the ZnO/PI/carbon fiber structure: (a) Rayleigh wave modes with λ = 64 μm and λ = 160 μm and (b) reflection spectra S_11_ of devices with λ = 64, 10, and 160 μm. Simulated patterns of (c) S_21_ spectrum of the electromagnetic resonator (the corresponding coupled SAW has a wavelength of 64 μm) and (d) profile of surface current density at the resonance (the corresponding coupled SAW has a wavelength of 64 μm).

FEA methods were used to investigate the Rayleigh wave modes and reflection spectra of SAW devices based on ZnO/PI/carbon fibers. Figure 3a displays the surface vibration modes of Rayleigh waves with wavelength of 64 and 160 μm. Since the Young’s modulus of the carbon fiber (97–228 GPa)^29^ is much larger than that of PI (∼2.5 GPa), the acoustic wave-induced mechanical energy is largely confined within the ZnO/PI structure. As the wavelength is increased and becomes comparable to the thickness of the trilayer structure, more energy becomes dissipated into the carbon fiber substrate as shown in Figure 3a. Simulation results present a similar changing trend of R_0_ frequency with increasing wavelength to those obtained from the experiments (Figure 3b). There is a minor divergence between experimental and simulation results (comparing the results shown in Figures 2b and 3b), which could be explained by the following reasons: (a) the chosen material parameters were obtained from those reported in the literature;^30−32^ (b) periodic boundary conditions were applied during the simulation, and (c) only one pair of IDT fingers were chosen during the simulation.

We also simulated the electromagnetic behavior of the device with a wavelength of 64 μm using a commercially available simulator. Figure 3c shows the transmission spectrum S_21_ of the device within a frequency range of 1–10 GHz, where the sharp dips at 4.6 and 8.1 GHz indicate two resonance modes. Here, the electric field is along the electrodes inducing electric polarization on the opposite bonding pads, which results in a circulating current pattern at 4.6 GHz as shown in Figure 3d. The electromagnetic signal is dissipated in the device at this frequency due to the induced current. A higher order resonance at 8.1 GHz results in a different pattern of circulating current as shown in Figure 3d. However, the resonance at 4.6 GHz is stronger than that at 8.1 GHz as the dip magnitude of the resonance is larger as observed in Figure 3c. Thus, we used this 4.6 GHz resonance for the metamaterial sensing work.

### Demonstration of Liquid Temperature Control Using the Integrated Platform

Precise temperature control of droplets is often desired for biosensors and bioreactors requiring biomolecular functionalization.^33^ The SAW devices can be used to increase and maintain the temperature of the liquid samples placed in the functional region of the sensor above the environmental temperature. The temperature rise in the liquid mainly results from an acousto-thermal heating phenomenon,^34^ depending on the input energy density of the acoustic waves and the energy dissipation into the liquid (mainly determined by the intrinsic properties of the liquid and its volume). Compared to the Al foil substrate, which we previously reported for use in the flexible SAW devices,^35^ the woven carbon fiber cloth substrate (which is polymer matrix based) has a relatively lower thermal conductivity on the order of 1–10 W/m·K.^36^ Together with the PI film between the ZnO layer and the carbon fiber substrate having an even smaller thermal conductivity of 0.12 W/m·K, most of the acoustic heat has been confined on the surface of the SAW device.

**Figure 4:**
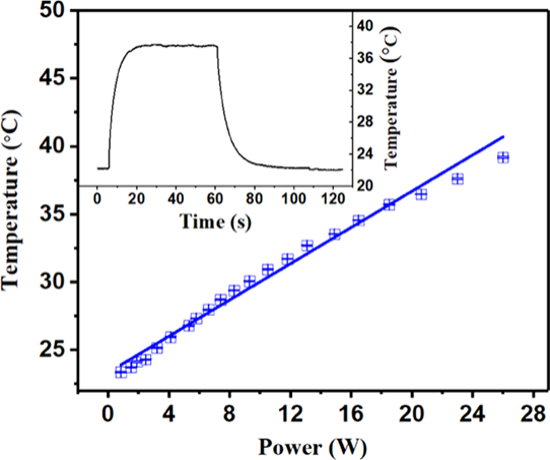
Measured average temperatures of a 5 μL distilled water droplet on top of the SAW device with increasing input power. The inset shows that the average temperature is controlled by the input power (23 W) over time.

We used the setup schematically shown in Figure 1e to measure the temperature of a droplet while the SAW device was activated. As a proof-of-concept demonstration, Figure 4 shows the average temperature of a 5 μL distilled water droplet on top of the SAW device with a wavelength of 160 μm controlled by the input SAW power. The obtained temperature readings are changed according to the following relationship with the applied power: *T* = 23.34 (°C) + 0.67 *P* (W), in which *T* is the droplet temperature and *P* is the input power applied to the IDTs at 12.33 MHz (Sezawa mode wave). The inset of Figure 4 displays an example of a heating cycle. The temperature was increased immediately after the power was applied, taking ∼10 s to reach the set value of 37.5 °C. Then, it was maintained at the set temperature for 1 min with a minor fluctuation of 0.1 °C. Clearly, SAW devices can be used to precisely control the liquid temperature, which can meet the requirements of biological processes. Besides, the temperature of the backside of the device (i.e., the carbon fiber surface) has been simulated using the FEA simulations for checking the biological safety factors. Assuming the environmental temperature is around 20 °C, the backside temperature has not been above 26 °C when the liquid above is maintained at 37 °C (see Figure SI1. a,b).

## Multiple Sensing Functions Based on the Integrated Platform

### UV Sensing Using SAW

**Figure 5:**
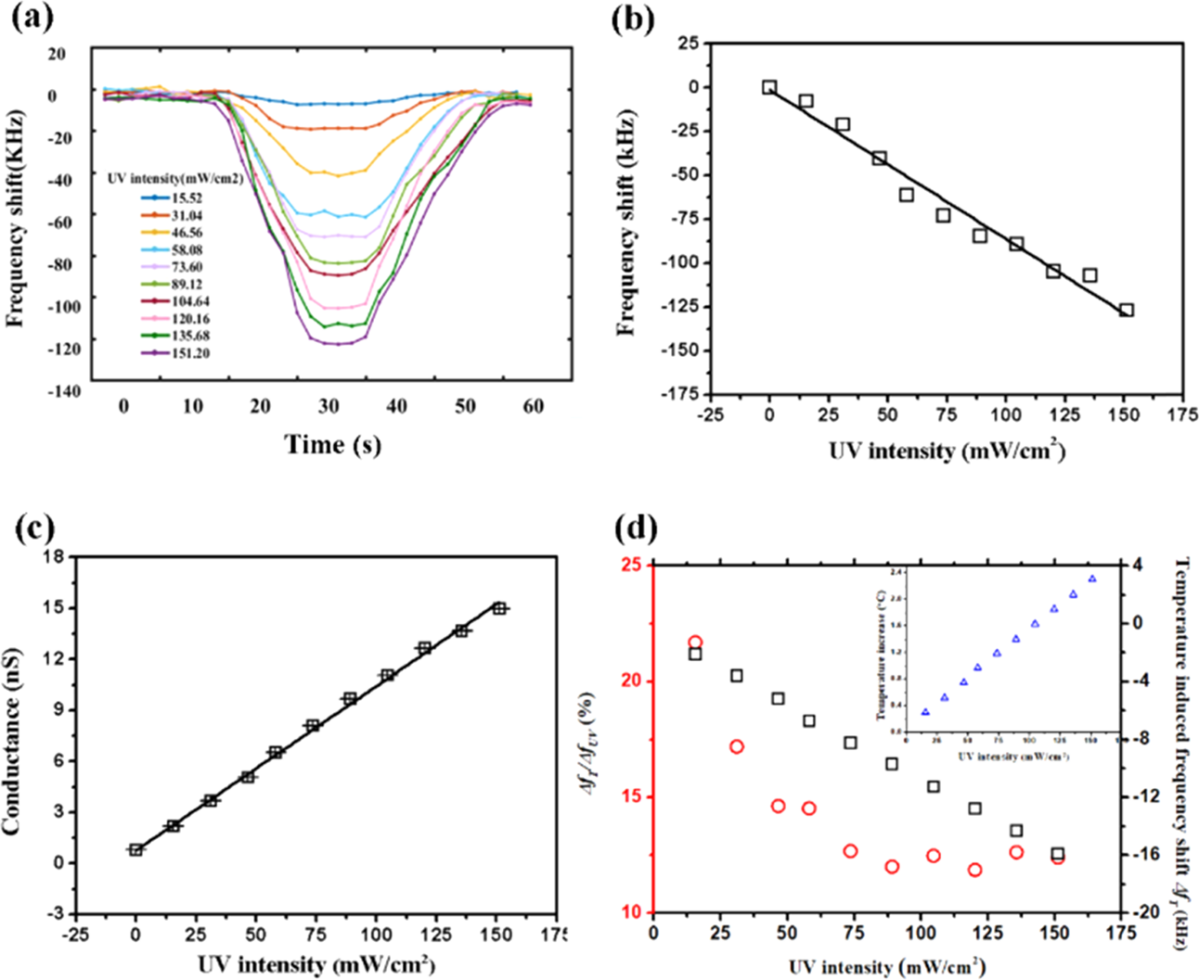
(a) Real-time frequency shift of the SAW UV sensor with a wavelength of 64 μm under UV light. (b) Total frequency shift varying with the UV intensity. (c) Sheet conductance varying with the UV intensity. (d) Temperature-change-induced frequency shift Δ*f*_T_ and the ratio between Δ*f*_T_ and the total shift varying with the UV intensity. The inset shows the temperature increase with the UV intensity.

The SAW device with a wavelength of 64 μm was used for demonstration of sensing functions such as UV sensing. We used the setup schematically shown in Figure 1d to measure the shift in resonant frequency of the SAW device under the UV exposure. As shown in Figure 5a, the device was exposed to the UV light with different controlled intensities (from 0 mW/cm^2^ to 151.2 mW/cm^2^) at durations of 20–40 s and then kept in the dark environment for another 20 s until the external UV irradiation influence disappeared, while the resonant frequency shift was continuously recorded for the whole process. As the device was exposed to the UV light, the frequency shift of the R_0_ mode was increased linearly for the first 10–15 s and then saturated at the corresponding intensity values until UV light was switched off. Afterward, the frequency shift was decreased to zero as the device recovered to the equilibrium state. Figure 5b shows that there is a linear relationship between the frequency shift and UV intensity, which produces an estimated sensitivity of 0.85 kHz/(mW/cm^2^). Considering that the initial frequency is 14.95 MHz, the sensitivity can also be written as 56.86 ppm/(mW/cm^2^).

According to eq 1, the frequency shift caused by the UV light is mainly composed of two parts: i.e., (a) from the conductivity change of ZnO thin films; and (b) from the increase of the temperature. For the frequency shift due to the changes of conductivity, the following equation is generally applied^37,38^
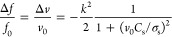
4where *k*^2^ is the coupling coefficient, *C*_s_ is the capacitance per unit length of the surface, and σ_s_ the sheet conductivity. By measuring the current–voltage (*I*–*V*) curves of the device under different intensities of the UV illumination (see Figure SI2 in the Supporting Information), the obtained sheet conductance *G*_s_ is shown in Figure 5c, and the readings increase with the UV intensity. As σ_s_ is proportional to *G*_s_, the sheet conductivity is also increased with the UV intensity, thus contributing to the increase of the total frequency shift.

However, thermal heating effect can also be generated in the device during the UV illumination due to the actuation of SAW and the low thermal conductivity of the PI-coated carbon fiber composites. This will surely change the shift of the frequency. We have also measured the temperature of the device as a function of UV exposure duration. The surface temperature rise was 0.3–2.3 K during the 20 s exposure at different UV intensities (Figure 5d). To evaluate the temperature-induced frequency shift, the temperature coefficient of frequency (TCF) of the same SAW device was measured and calculated, and the obtained reading was 465 ppm/K (with the initial frequency *f*_0_ = 14.95 MHz). The frequency shift Δ*f*_T_ can be calculated using the following equation
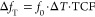
5where Δ*T* is the change of temperature. Therefore, the temperature-induced frequency shift was estimated to be −2 to −16 kHz, which contributes to less than 25% of the total frequency shift as shown in Figure 5d. Besides, this fraction was decreased as the UV intensity was increased and saturated at 12%. In addition to temperature, humidity as another key environmental parameter can also affect the UV-sensing performance of SAW sensors. We have previously explored this effect for Al-foil-based flexible SAW sensors and explained how the measurements can be decoupled.^37,38^

Our experimental results showed that the SAW resonant frequency can be used for UV sensing and indicated the conductivity change of the ZnO thin film is dominant in the physical mechanism.

### Glucose Concentration Monitoring Using the Electromagnetic Resonator

**Figure 6:**
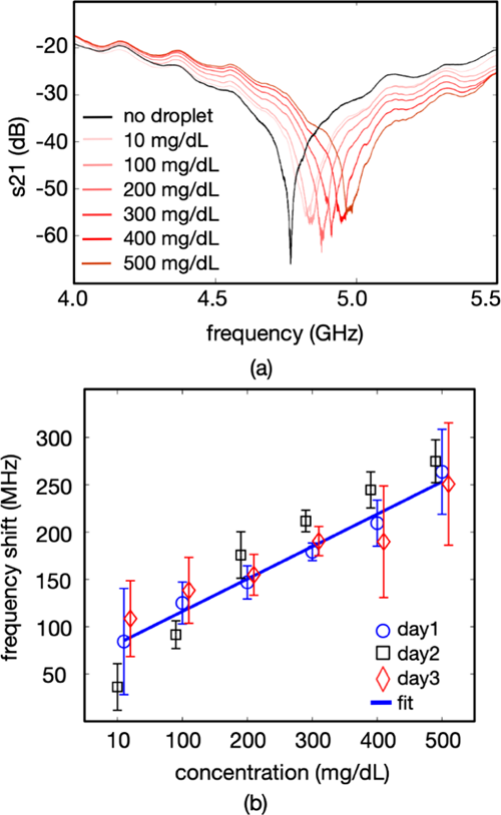
(a) S_21_ spectra of the device with droplets with varying concentrations of glucose and (b) frequency shift of the device with glucose concentration, measured on three different days. The concentration values for each day were the same at 10, 100, 200, 300, 400, and 500 mg/dL. The markers in the figures are shifted slightly in the horizontal direction for better readability.

The same SAW device (with the wavelength of 64 μm) was further used as the metamaterial device to measure glucose concentrations in a droplet of deionized water with a volume of 0.5 μL placed directly on top of the IDTs (see Figure 1c for the schematics of the experimental setup). We kept the droplet at the exactly same location on the device with a position error of less than 0.2 mm using the IDT itself as the marker under the video camera. We then varied the concentrations of glucose within a range of 10–500 mg/dL and also washed the surface with deionized water between each measurement to clean the residues. Figure 6a shows an exemplary set of recorded S_21_ spectra at different glucose concentrations. We repeated each measurement at a particular glucose concentration for 10 times and repeated the measurement protocol on three different days. Figure 6b shows the variation of the resonant frequency with the concentration of glucose, where the error bars represent the standard error of the mean values. The resonant frequency of the metamaterial device increases with the concentration of glucose. This is expected since the permittivity of a droplet of glucose solution decreases with increased concentration of glucose.^22^ We observed a linear decrease in resonant frequency within the measurement range with a sensitivity of 0.34 MHz/(mg/dL). This level allows measurement of glucose with a resolution of 3 μg/dL with a frequency resolution of 1 kHz at the measurement band.

## Conclusions

A flexible and integrated platform of acoustic waves and electromagnetic metamaterials based on polyimide-coated woven carbon fibers was proposed in this work for potential application in bioassays and multifunction sensing. The designed platform was based on a SAW device, where the acoustic wave was agitated to control the temperature of a liquid droplet placed in the functional area and was also used as a UV sensor with the sensitivity of 56.86 ppm/(mW/cm^2^). Meanwhile, the same device presented excellent performance in glucose concentration monitoring when it worked as an electromagnetic metamaterial device, giving a sensitivity of 0.34 MHz/(mg/dL). Our integrated platform has shown its capability for versatile sensing functions in a liquid environment as well as the capability to simulate the biological incubating conditions.

## Supporting Information Available

FEA simulation to evaluate the heat transfer in the SAW device (Figure SI1); *I*–*V* curves of the SAW device with the wavelength of 64 μm under different intensities of the UV illumination (Figure SI2) (PDF)
