# Supplementary material for: Flexible and Integrated Sensing Platform of Acoustic Waves and Metamaterials based on Polyimide-Coated Woven Carbon Fibers
Source: ACS Sens. 2020 Jul 20;5(8):2563–9. doi: 10.1021/acssensors.0c00948 (PMC8009594; doi:10.1021/acssensors.0c00948)
Supplement: Supplementary file 1 — se0c00948_si_001.pdf [file se0c00948_si_001.pdf]

Supporting Information

for

Flexible and integrated sensing platform of acoustic waves and metamaterials based on polyimide coated woven carbon fibers

Ran Tao,[1,2] Shahrzad Zahertar,[2] Hamdi Torun,[2] Yi Ru Liu,[3] Meng Wang,[3] Yuchao Lu,[4] Jing Ting Luo,[1] Jethro Vernon,[2] Richard Binns,[2] Yang He,[4] Kai Tao,[4] Qiang Wu,[2] Hong Long Chang,[4] Yong Qing Fu[2,*]

1. Shenzhen Key Laboratory of Advanced Thin Films and Applications, College of Physics and Optoelectronic Engineering, Shenzhen University, Shenzhen, 518060, P. R. China

2. Faculty of Engineering and Environment, Northumbria University, Newcastle upon Tyne NE1 8ST, UK

3. China-EU Institute for Clean and Renewable Energy, Huazhong University of Science and Technology, Wuhan, 430074, P.R. China

4. Key Laboratory of Micro and Nano Systems for Aerospace, Ministry of Education, Northwestern Polytechnical University, Xi'an 710072, PR China

*Corresponding author, Prof. Richard Yongqing Fu, Email: Richard.fu@northumbria.ac.uk

FEA simulation to evaluate the heat transfer in the SAW device.

We have used 2D FEA models to study the heat distribution inside the multi-layer structure using the module of Heat Transfer in Solids in COMSOL. The external temperature of the top side is considered to be the set temperature of the liquid droplet, which is assumed to be 37 °C, while the backside (carbon fiber surface) is assumed to be 20 °C. Figure SI1 shows the temperature distribution of the whole SAW structure, from which we can tell that the backside temperature does not exceed 26 °C.

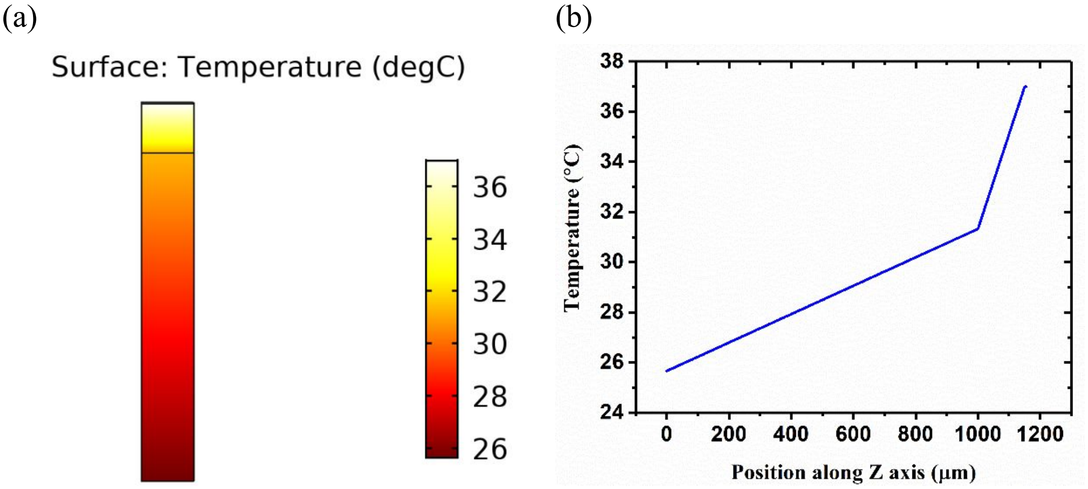

Figure SI1. (a) Cross-section temperature distribution and (b) Temperature along the Z axis of the multi-layer structure of SAWs when the temperature of the liquid is 37 °C.

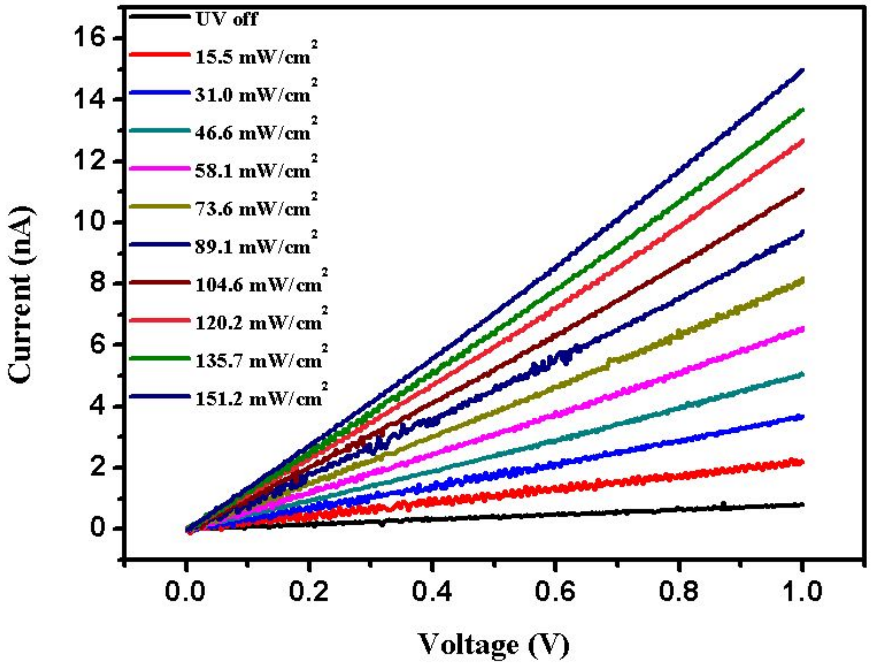

Figure SI2. Current-voltage (I-V) curves of the SAW device with the wavelength of 64 μm under different intensities of the UV illumination
